# 2-[(2,6-Diethyl­phen­yl)imino­meth­yl]-*N*-(2-methoxy­phen­yl)aniline

**DOI:** 10.1107/S1600536809037969

**Published:** 2009-09-26

**Authors:** Qing Su, Qiao-Lin Wu, Ling Ye, Ying Mu

**Affiliations:** a School of Chemistry, Jilin University, Changchun 130012, People’s Republic of China; b State Key Laboratory of Supramolecular Structure and Materials, School of Chemistry, Jilin University, Changchun 130012, People’s Republic of China

## Abstract

The title anilide–imine compound, C_24_H_26_N_2_O, features an intra­molecular N—H⋯N hydrogen bond involving the imine and anilide groups to generate an *S*(6) ring motif. The mol­ecule displays an *E* configuration about the imine C=N double bond, with the dihedral angle between the two benzene rings being 86.5°. The packing is stabilized by three different C—H⋯π inter­actions.

## Related literature

For related background on anilido–imine complexes, see: Liu *et al.* (2005 ▶, 2006 ▶); Ren *et al.* (2007 ▶); Su *et al.* (2007 ▶); Yao *et al.* (2008 ▶); Wang *et al.* (2006 ▶). For hydrogen-bond motifs, see: Bernstein *et al.* (1995 ▶).
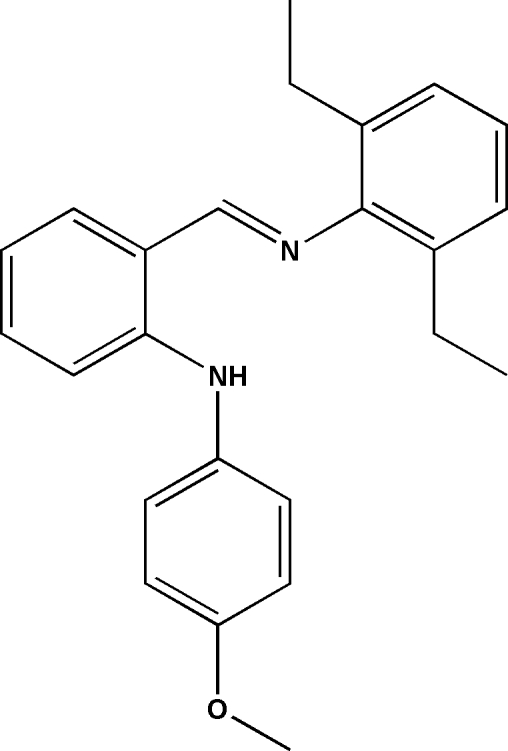


## Experimental

### 

#### Crystal data

C_24_H_26_N_2_O
*M* *_r_* = 358.47
Monoclinic, 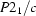

*a* = 12.930 (3) Å
*b* = 7.4757 (15) Å
*c* = 21.303 (4) Å
β = 97.88 (3)°
*V* = 2039.7 (7) Å^3^
*Z* = 4
Mo *K*α radiation
μ = 0.07 mm^−1^
*T* = 295 K
0.44 × 0.40 × 0.19 mm

#### Data collection

Rigaku R-AXIS RAPID diffractometer
Absorption correction: multi-scan (*SADABS*; Bruker, 2001 ▶) *T*_min_ = 0.969, *T*_max_ = 0.986
19406 measured reflections
4670 independent reflections
3515 reflections with *I* > 2σ(*I*)
*R*_int_ = 0.035

#### Refinement

*R*[*F* ^2^ > 2σ(*F* ^2^)] = 0.040
*wR*(*F* ^2^) = 0.108
*S* = 1.06
4670 reflections
252 parameters
H atoms treated by a mixture of independent and constrained refinement
Δρ_max_ = 0.19 e Å^−3^
Δρ_min_ = −0.17 e Å^−3^

### 

Data collection: *RAPID-AUTO* (Rigaku, 1998 ▶); cell refinement: *RAPID-AUTO*; data reduction: *CrystalStructure* (Rigaku/MSC, 2002 ▶); program(s) used to solve structure: *SHELXS97* (Sheldrick, 2008 ▶); program(s) used to refine structure: *SHELXL97* (Sheldrick, 2008 ▶); molecular graphics: *XP* in *SHELXTL* (Sheldrick, 2008 ▶); software used to prepare material for publication: *SHELXTL*.

## Supplementary Material

Crystal structure: contains datablocks global, I. DOI: 10.1107/S1600536809037969/fl2258sup1.cif

Structure factors: contains datablocks I. DOI: 10.1107/S1600536809037969/fl2258Isup2.hkl

Additional supplementary materials:  crystallographic information; 3D view; checkCIF report

## Figures and Tables

**Table 1:** Hydrogen-bond geometry (Å, °)

*D*—H⋯*A*	*D*—H	H⋯*A*	*D*⋯*A*	*D*—H⋯*A*
N1—H1⋯N2	0.902 (14)	1.976 (15)	2.7126 (15)	137.8 (12)
C5—H5⋯*Cg*2^i^	0.93	2.79	3.5296 (8)	137
C10—H10⋯*Cg*1^ii^	0.93	2.84	3.7651 (6)	176
C16—H16*A*⋯*Cg*3^iii^	0.97	2.82	3.6614 (7)	146

Symmetry codes: (i) 
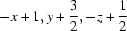
; (ii) 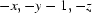; (iii) 
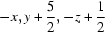
. *Cg*1, *Cg*2 and *Cg*3 are the centroids the C8–C13, C18–C23 and C1–C6 rings, respectively.

**Fig. 1.**
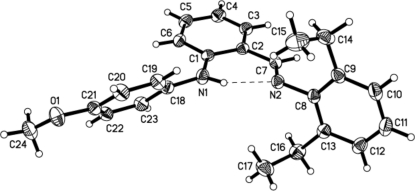
View of the molecule of (I) showing the atom labelling scheme. Displacement ellipsoids are drawn at the 30% probability level. The dashed line indicates an intramolecular hydrogen bond.

**Fig. 2.**
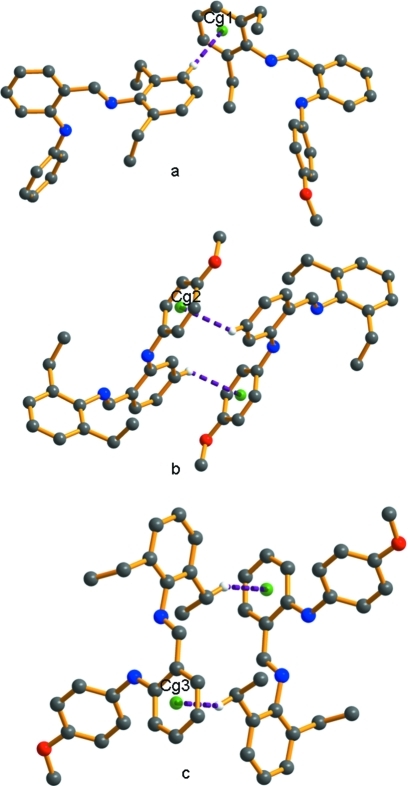
Packing of the molecule of (I) showing the different C—H···π interactions. Cg1: the centroid of the benzene ring (C8—C13); Cg2: the centroid of the benzene ring (C18—C23); Cg3: the centroid of the benzene ring (C1—C6). C10···Cg1 = 3.7651 (6) Å, C5···Cg2 = 3.5296 (8) Å, and C16···Cg3 = 3.6614 (7) Å. Only the hydrogen atoms involved in supramolecular interactions are shown for clarity. The green dots are the centers of the aromatic rings.

## supplementary crystallographic information

### Comment

A chelating anilide-imine compound has recently attracted increasing attention because it has a similar framwork  and combination of  steric and electronic characterics found in  β-diketiminate and salicyaldiminato ligands which have been extensively researched in coordination chemistry and catalysis. (Wang *et al.*, 2006) We have recently reported the luminescent properties of a series of zinc(II) (Su *et al.*, 2007), aluminium(III) (Liu *et al.*, 2005; 2006) and boron(III) (Ren *et al.*, 2007) complexes with chelating anilido-imine ligands and catalytical properties of aluminium(III) (Yao *et al.*, 2008) for the polymerization of ε-caprolactone. Good results have been obtained. As a part of our study, the preparation and crystal structure of the new anilido-imine title compound (I) (Fig. 1), is reported.

The bond lengths and angles are within normal ranges. The C7=N2 [1.2730 (15) Å] bond length is comparable to those found in similar anilido-imine compounds, such as {2-[(2,6-diethyl-phenylimino)-methyl]-phenyl}- (2,4-dimethyl-quinolin-7-yl)-amine [1.267 (4), 1.275 (4) Å] (Su *et al.*, 2007) and {2-[(2,6-methyl-phenylimino)-methyl]-phenyl}- (2,4-dimethyl-quinolin-7-yl)-amine [1.271 (2) Å] (Su *et al.*, 2007). The molecule adopts an *E* configuration about the central C=N bond. The dihedral angles between the central benzene ring (C1—C6) and the two outer benzene rings of the anilido-imine compound are 86.5° (C8—C13) and 54.2° (C18—C23). The dihedral angle between the C8—C13 and C18—C23 phenyl rings is 113.4°. An intramolecular N1—H1···N2 hydrogen bond forms a six-membered ring, generating an S(6) motif (Bernstein *et al.*, 1995).

In the packing of the crystal, there exists three different types of C—H···π interactions (Fig.2 and Table1). The C—H···π interactions involving H10 form chains (Fig.2a). The additional C—H···π interactions through H5 and H16*a* interlink these chains (Fig.2b, and Fig.2c).

### Experimental

Preligand (2,6-Diethyl-phenyl)-(2-fluoro-benzylidene)-amine [*ortho*-C_6_H_4_(CH=NC_6_H_3_Et_2_-2,6)] was synthesized according to a literature method (Su *et al.*, 2007). A solution of *n*BuLi (1.60 mol/*L*, 9.2 mmol) in *n*-hexane was added to a solution of 4-methoxy-phenylamine (1.14 g, 9.2 mmol) in THF (20 ml) at -78 °C. The mixture was allowed to warm to room temperature and stirred for additional 4 h. The resulting solution was transferred into a solution of *ortho*-C_6_H_4_(CH=NC_6_H_3_Et_2_-2,6) (2.36 g, 9.2 mmol) in THF at 25 °C. After stirring for two days, the reaction was quenched with H_2_O (20 ml). The organic phase was evaporated to dryness to give the crude product as a brown oil that was purified by column chromatography on silica gel with ethyl acetate/ petroleum ether (1:34 in volume) as eluent. The pure product as yellow crystals (1.72 g, 52%) suitable for data collection were obtained after concentrating the solution. Anal. Calcd. for C_24_H_26_N_2_O (358.48): C 80.41, H 7.36, N 7.81; Found: C 80.26, H 7.25, N 7.90%. ^1^H NMR (500 MHz, CDCl_3_, 298 K) δ (p.p.m.): 1.20 (t, 2 x 3H, J = 7.5 Hz, C*H*_3_CH_2_), 2.60 (q, 2 x 2H, J = 7.5 Hz, CH_3_C*H*_2_), 3.86 (s, 3H, O—C*H*_3_), 6.80 (t, 1H, J = 6.5 Hz), 6.95 (d, 2H, J = 7.5 Hz), 7.10 (d, 1H, J = 6.0 Hz), 7.16 (br, 2H), 7.20 (d, 1H, J = 9.0 Hz), 7.28 (d, 3H, J = 3.5 Hz), 7.37 (d, 1H, J = 7.0 Hz), 8.76 (s, 1H, C*H*=N), 10.99 (br, 1H, N*H*).

### Refinement

The C-bound H atoms were positioned geometrically with C—H = 0.93 (aromatic and imine carbon), 0.97 (methylene) and 0.96 (methyl) Å, and allowed to ride on their parent atoms in the riding model approximation with *U*_iso_(H) = 1.2 (1.5 for methyl) *U*_eq_(C). The atom H1 was located in a difference Fourier map and refined isotropically.

### Crystal data

**Table:**

C_24_H_26_N_2_O	*F*(000) = 768
*M**_r_* = 358.47	*D*_x_ = 1.167 Mg m^−^^3^
Monoclinic, *P*2_1_/*c*	Melting point: not measured K
Hall symbol: -P 2ybc	Mo *K*α radiation, λ = 0.71073 Å
*a* = 12.930 (3) Å	Cell parameters from 14731 reflections
*b* = 7.4757 (15) Å	θ = 3.2–27.5°
*c* = 21.303 (4) Å	µ = 0.07 mm^−^^1^
β = 97.88 (3)°	*T* = 295 K
*V* = 2039.7 (7) Å^3^	Block, yellow
*Z* = 4	0.44 × 0.40 × 0.19 mm

### Data collection

**Table:**

Rigaku R-AXIS RAPID diffractometer	4670 independent reflections
Radiation source: fine-focus sealed tube	3515 reflections with *I* > 2σ(*I*)
graphite	*R*_int_ = 0.035
ω scans	θ_max_ = 27.5°, θ_min_ = 3.2°
Absorption correction: multi-scan (*SADABS*; Bruker, 2001)	*h* = −16→16
*T*_min_ = 0.969, *T*_max_ = 0.986	*k* = −9→9
19406 measured reflections	*l* = −27→27

### Refinement

**Table:**

Refinement on *F*^2^	Secondary atom site location: difference Fourier map
Least-squares matrix: full	Hydrogen site location: inferred from neighbouring sites
*R*[*F*^2^ > 2σ(*F*^2^)] = 0.040	H atoms treated by a mixture of independent and constrained refinement
*wR*(*F*^2^) = 0.108	*w* = 1/[σ^2^(*F*_o_^2^) + (0.0487*P*)^2^ + 0.2475*P*] where *P* = (*F*_o_^2^ + 2*F*_c_^2^)/3
*S* = 1.06	(Δ/σ)_max_ < 0.001
4670 reflections	Δρ_max_ = 0.19 e Å^−^^3^
252 parameters	Δρ_min_ = −0.17 e Å^−^^3^
0 restraints	Extinction correction: *SHELXL97* (Sheldrick, 2008), Fc^*^=kFc[1+0.001xFc^2^λ^3^/sin(2θ)]^-1/4^
Primary atom site location: structure-invariant direct methods	Extinction coefficient: 0.0251 (17)

### Special details

**Table:**

Experimental. (See detailed section in the paper)
Geometry. All e.s.d.'s (except the e.s.d. in the dihedral angle between two l.s. planes) are estimated using the full covariance matrix. The cell e.s.d.'s are taken into account individually in the estimation of e.s.d.'s in distances, angles and torsion angles; correlations between e.s.d.'s in cell parameters are only used when they are defined by crystal symmetry. An approximate (isotropic) treatment of cell e.s.d.'s is used for estimating e.s.d.'s involving l.s. planes.
Refinement. Refinement of *F*^2^ against ALL reflections. The weighted *R*-factor *wR* and goodness of fit *S* are based on *F*^2^, conventional *R*-factors *R* are based on *F*, with *F* set to zero for negative *F*^2^. The threshold expression of *F*^2^ > σ(*F*^2^) is used only for calculating *R*-factors(gt) *etc*. and is not relevant to the choice of reflections for refinement. *R*-factors based on *F*^2^ are statistically about twice as large as those based on *F*, and *R*- factors based on ALL data will be even larger.

### Fractional atomic coordinates and isotropic or equivalent isotropic displacement parameters (Å^2^)

**Table:**

	*x*	*y*	*z*	*U*_iso_*/*U*_eq_	
O1	0.52847 (8)	1.56960 (13)	0.17089 (5)	0.0608 (3)	
N1	0.28321 (8)	0.98441 (15)	0.08469 (5)	0.0460 (3)	
N2	0.12969 (8)	0.75355 (13)	0.10555 (5)	0.0405 (2)	
C1	0.28918 (8)	0.88995 (15)	0.03007 (5)	0.0363 (3)	
C2	0.22193 (8)	0.74272 (15)	0.01420 (5)	0.0367 (3)	
C3	0.22900 (9)	0.64858 (17)	−0.04164 (6)	0.0436 (3)	
H3	0.1840	0.5530	−0.0523	0.052*	
C4	0.30038 (10)	0.69250 (19)	−0.08147 (6)	0.0507 (3)	
H4	0.3037	0.6282	−0.1185	0.061*	
C5	0.36708 (10)	0.83456 (19)	−0.06505 (6)	0.0500 (3)	
H5	0.4164	0.8647	−0.0912	0.060*	
C6	0.36198 (9)	0.93198 (17)	−0.01092 (6)	0.0443 (3)	
H6	0.4075	1.0274	−0.0012	0.053*	
C7	0.14318 (9)	0.68530 (16)	0.05253 (5)	0.0390 (3)	
H7	0.0994	0.5918	0.0372	0.047*	
C8	0.04576 (9)	0.68824 (16)	0.13614 (5)	0.0392 (3)	
C9	0.06378 (10)	0.54991 (16)	0.18027 (6)	0.0455 (3)	
C10	−0.01957 (12)	0.49231 (18)	0.21017 (7)	0.0554 (4)	
H10	−0.0096	0.3997	0.2395	0.067*	
C11	−0.11638 (12)	0.5703 (2)	0.19697 (7)	0.0582 (4)	
H11	−0.1714	0.5293	0.2170	0.070*	
C12	−0.13204 (10)	0.7088 (2)	0.15432 (7)	0.0535 (4)	
H12	−0.1978	0.7610	0.1461	0.064*	
C13	−0.05162 (9)	0.77272 (17)	0.12316 (6)	0.0439 (3)	
C14	0.17116 (13)	0.4706 (2)	0.19727 (7)	0.0612 (4)	
H14A	0.2026	0.4539	0.1589	0.073*	
H14B	0.1649	0.3540	0.2164	0.073*	
C15	0.24158 (13)	0.5868 (3)	0.24255 (9)	0.0841 (5)	
H15A	0.2506	0.7006	0.2231	0.126*	
H15B	0.3082	0.5296	0.2528	0.126*	
H15C	0.2107	0.6041	0.2806	0.126*	
C16	−0.06704 (11)	0.9317 (2)	0.07983 (7)	0.0566 (4)	
H16A	−0.1412	0.9560	0.0700	0.068*	
H16B	−0.0408	0.9033	0.0405	0.068*	
C17	−0.01226 (13)	1.0986 (2)	0.10827 (9)	0.0700 (4)	
H17A	−0.0415	1.1328	0.1455	0.105*	
H17B	−0.0215	1.1940	0.0779	0.105*	
H17C	0.0609	1.0744	0.1194	0.105*	
C18	0.34258 (9)	1.13770 (16)	0.10489 (6)	0.0398 (3)	
C19	0.39696 (10)	1.14282 (17)	0.16584 (6)	0.0452 (3)	
H19	0.3931	1.0463	0.1929	0.054*	
C20	0.45650 (10)	1.28936 (18)	0.18645 (6)	0.0487 (3)	
H20	0.4920	1.2915	0.2275	0.058*	
C21	0.46397 (9)	1.43359 (17)	0.14663 (6)	0.0434 (3)	
C22	0.40796 (10)	1.43273 (17)	0.08638 (6)	0.0455 (3)	
H22	0.4112	1.5299	0.0595	0.055*	
C23	0.34714 (9)	1.28581 (17)	0.06663 (6)	0.0445 (3)	
H23	0.3083	1.2867	0.0265	0.053*	
C24	0.54257 (13)	1.7158 (2)	0.13088 (9)	0.0701 (5)	
H24A	0.5698	1.6736	0.0938	0.105*	
H24B	0.5908	1.7992	0.1531	0.105*	
H24C	0.4767	1.7739	0.1185	0.105*	
H1	0.2406 (11)	0.937 (2)	0.1104 (7)	0.056 (4)*	

### Atomic displacement parameters (Å^2^)

**Table:**

	*U*^11^	*U*^22^	*U*^33^	*U*^12^	*U*^13^	*U*^23^
O1	0.0714 (6)	0.0509 (6)	0.0578 (6)	−0.0154 (5)	0.0004 (5)	−0.0115 (5)
N1	0.0485 (6)	0.0493 (6)	0.0423 (6)	−0.0118 (5)	0.0134 (5)	−0.0073 (5)
N2	0.0442 (5)	0.0395 (5)	0.0387 (5)	−0.0029 (4)	0.0090 (4)	0.0020 (4)
C1	0.0339 (5)	0.0391 (6)	0.0354 (6)	0.0038 (4)	0.0026 (4)	0.0005 (5)
C2	0.0359 (5)	0.0382 (6)	0.0355 (6)	0.0032 (5)	0.0035 (5)	0.0024 (5)
C3	0.0442 (6)	0.0432 (7)	0.0432 (6)	0.0000 (5)	0.0051 (5)	−0.0054 (5)
C4	0.0533 (7)	0.0565 (8)	0.0442 (7)	0.0027 (6)	0.0135 (6)	−0.0108 (6)
C5	0.0448 (7)	0.0593 (8)	0.0491 (7)	0.0014 (6)	0.0179 (6)	−0.0019 (6)
C6	0.0372 (6)	0.0471 (7)	0.0498 (7)	−0.0025 (5)	0.0100 (5)	−0.0018 (6)
C7	0.0407 (6)	0.0356 (6)	0.0403 (6)	−0.0022 (5)	0.0040 (5)	0.0010 (5)
C8	0.0452 (6)	0.0371 (6)	0.0364 (6)	−0.0069 (5)	0.0096 (5)	−0.0048 (5)
C9	0.0601 (7)	0.0365 (6)	0.0422 (7)	−0.0020 (5)	0.0155 (6)	−0.0023 (5)
C10	0.0805 (10)	0.0415 (7)	0.0488 (7)	−0.0124 (7)	0.0252 (7)	−0.0025 (6)
C11	0.0649 (9)	0.0601 (9)	0.0549 (8)	−0.0248 (7)	0.0274 (7)	−0.0164 (7)
C12	0.0436 (7)	0.0638 (9)	0.0543 (8)	−0.0100 (6)	0.0105 (6)	−0.0166 (7)
C13	0.0430 (6)	0.0475 (7)	0.0403 (6)	−0.0063 (5)	0.0027 (5)	−0.0077 (5)
C14	0.0806 (10)	0.0491 (8)	0.0576 (9)	0.0180 (7)	0.0223 (8)	0.0147 (7)
C15	0.0681 (10)	0.0977 (14)	0.0828 (12)	0.0204 (10)	−0.0036 (9)	0.0036 (11)
C16	0.0490 (7)	0.0647 (9)	0.0534 (8)	0.0063 (6)	−0.0028 (6)	0.0061 (7)
C17	0.0731 (10)	0.0509 (9)	0.0823 (11)	0.0074 (7)	−0.0021 (8)	0.0104 (8)
C18	0.0384 (6)	0.0414 (6)	0.0404 (6)	−0.0009 (5)	0.0078 (5)	−0.0056 (5)
C19	0.0549 (7)	0.0443 (7)	0.0369 (6)	−0.0011 (6)	0.0081 (5)	0.0005 (5)
C20	0.0579 (8)	0.0530 (8)	0.0338 (6)	−0.0018 (6)	0.0009 (5)	−0.0055 (5)
C21	0.0444 (6)	0.0408 (7)	0.0447 (7)	−0.0008 (5)	0.0057 (5)	−0.0096 (5)
C22	0.0494 (7)	0.0398 (7)	0.0467 (7)	0.0027 (5)	0.0043 (5)	0.0024 (5)
C23	0.0440 (6)	0.0467 (7)	0.0404 (6)	0.0017 (5)	−0.0026 (5)	0.0000 (5)
C24	0.0739 (10)	0.0446 (8)	0.0899 (12)	−0.0133 (7)	0.0053 (9)	−0.0044 (8)

### Geometric parameters (Å, °)

**Table:**

O1—C21	1.3704 (15)	C12—H12	0.9300
O1—C24	1.4130 (18)	C13—C16	1.5011 (19)
N1—C1	1.3723 (15)	C14—C15	1.508 (2)
N1—C18	1.4131 (16)	C14—H14A	0.9700
N1—H1	0.902 (14)	C14—H14B	0.9700
N2—C7	1.2730 (15)	C15—H15A	0.9600
N2—C8	1.4265 (15)	C15—H15B	0.9600
C1—C6	1.4045 (16)	C15—H15C	0.9600
C1—C2	1.4147 (16)	C16—C17	1.519 (2)
C2—C3	1.3959 (16)	C16—H16A	0.9700
C2—C7	1.4545 (16)	C16—H16B	0.9700
C3—C4	1.3765 (17)	C17—H17A	0.9600
C3—H3	0.9300	C17—H17B	0.9600
C4—C5	1.3824 (19)	C17—H17C	0.9600
C4—H4	0.9300	C18—C23	1.3809 (18)
C5—C6	1.3731 (18)	C18—C19	1.3898 (18)
C5—H5	0.9300	C19—C20	1.3758 (18)
C6—H6	0.9300	C19—H19	0.9300
C7—H7	0.9300	C20—C21	1.3833 (19)
C8—C9	1.3955 (17)	C20—H20	0.9300
C8—C13	1.4019 (17)	C21—C22	1.3846 (19)
C9—C10	1.3932 (18)	C22—C23	1.3824 (18)
C9—C14	1.507 (2)	C22—H22	0.9300
C10—C11	1.375 (2)	C23—H23	0.9300
C10—H10	0.9300	C24—H24A	0.9600
C11—C12	1.374 (2)	C24—H24B	0.9600
C11—H11	0.9300	C24—H24C	0.9600
C12—C13	1.3925 (18)		
			
C21—O1—C24	117.94 (11)	C15—C14—H14A	109.1
C1—N1—C18	125.73 (10)	C9—C14—H14B	109.1
C1—N1—H1	115.0 (9)	C15—C14—H14B	109.1
C18—N1—H1	119.1 (9)	H14A—C14—H14B	107.8
C7—N2—C8	118.26 (10)	C14—C15—H15A	109.5
N1—C1—C6	122.17 (11)	C14—C15—H15B	109.5
N1—C1—C2	119.91 (10)	H15A—C15—H15B	109.5
C6—C1—C2	117.90 (10)	C14—C15—H15C	109.5
C3—C2—C1	119.07 (10)	H15A—C15—H15C	109.5
C3—C2—C7	117.53 (11)	H15B—C15—H15C	109.5
C1—C2—C7	123.38 (10)	C13—C16—C17	112.94 (12)
C4—C3—C2	122.20 (12)	C13—C16—H16A	109.0
C4—C3—H3	118.9	C17—C16—H16A	109.0
C2—C3—H3	118.9	C13—C16—H16B	109.0
C3—C4—C5	118.36 (12)	C17—C16—H16B	109.0
C3—C4—H4	120.8	H16A—C16—H16B	107.8
C5—C4—H4	120.8	C16—C17—H17A	109.5
C6—C5—C4	121.32 (11)	C16—C17—H17B	109.5
C6—C5—H5	119.3	H17A—C17—H17B	109.5
C4—C5—H5	119.3	C16—C17—H17C	109.5
C5—C6—C1	121.12 (12)	H17A—C17—H17C	109.5
C5—C6—H6	119.4	H17B—C17—H17C	109.5
C1—C6—H6	119.4	C23—C18—C19	118.18 (11)
N2—C7—C2	124.84 (11)	C23—C18—N1	122.41 (11)
N2—C7—H7	117.6	C19—C18—N1	119.40 (11)
C2—C7—H7	117.6	C20—C19—C18	120.62 (12)
C9—C8—C13	121.95 (11)	C20—C19—H19	119.7
C9—C8—N2	119.61 (11)	C18—C19—H19	119.7
C13—C8—N2	118.34 (11)	C19—C20—C21	120.54 (12)
C10—C9—C8	117.96 (12)	C19—C20—H20	119.7
C10—C9—C14	120.89 (12)	C21—C20—H20	119.7
C8—C9—C14	121.10 (11)	O1—C21—C20	115.93 (11)
C11—C10—C9	121.01 (13)	O1—C21—C22	124.53 (12)
C11—C10—H10	119.5	C20—C21—C22	119.54 (12)
C9—C10—H10	119.5	C23—C22—C21	119.29 (12)
C12—C11—C10	120.16 (12)	C23—C22—H22	120.4
C12—C11—H11	119.9	C21—C22—H22	120.4
C10—C11—H11	119.9	C18—C23—C22	121.73 (12)
C11—C12—C13	121.45 (13)	C18—C23—H23	119.1
C11—C12—H12	119.3	C22—C23—H23	119.1
C13—C12—H12	119.3	O1—C24—H24A	109.5
C12—C13—C8	117.43 (13)	O1—C24—H24B	109.5
C12—C13—C16	121.32 (12)	H24A—C24—H24B	109.5
C8—C13—C16	121.18 (11)	O1—C24—H24C	109.5
C9—C14—C15	112.50 (13)	H24A—C24—H24C	109.5
C9—C14—H14A	109.1	H24B—C24—H24C	109.5

### Hydrogen-bond geometry (Å, °)

**Table:**

*D*—H···*A*	*D*—H	H···*A*	*D*···*A*	*D*—H···*A*
N1—H1···N2	0.902 (14)	1.976 (15)	2.7126 (15)	137.8 (12)
C5—H5···Cg2^i^	0.93	2.79	3.5296 (8)	137
C10—H10···Cg1^ii^	0.93	2.84	3.7651 (6)	176
C16—H16A···Cg3^iii^	0.97	2.82	3.6614 (7)	146

Symmetry codes:  (i) −*x*+1, *y*+3/2, −*z*+1/2; (ii) −*x*, −*y*−1, −*z*; (iii) −*x*, *y*+5/2, −*z*+1/2.
